# Dataset of measurements for the experimental CEA-beam benchmark structure subjected to one stochastic broadband excitation.

**DOI:** 10.1016/j.dib.2021.106798

**Published:** 2021-01-28

**Authors:** T. Roncen, J.-P. Lambelin, Y. Chantereau, J-J. Sinou

**Affiliations:** a CEA/CESTA, CS60001, 15 avenue des Salinières, 33116 Le Barp, France; b Laboratoire de Tribologie et Dynamique des Systèmes, UMR CNRS 5513, Ecole Centrale de Lyon, 36 avenue Guy de Collongue 69134 Ecully Cedex, France; c Institut Universitaire de France, 75005 Paris, France

**Keywords:** Nonlinear vibration, CEA-beam benchmark structure, Stochastic broadband excitation, Non-ideal boundary conditions

## Abstract

This data article comprises data to investigate the non-linear dynamic behavior of the CEA-beam benchmark structure subjected to one stochastic broadband excitation. Experiments have been performed on the CEA-CESTA laboratory. The data provided include the input Power Spectral Density for four levels of excitation and the associated output nonlinear dynamic behavior of the CEA-beam benchmark structure. All the results from this data will help researchers and engineers in proper analysis of hardening effect and the enlargement of the response peak due to one stochastic broadband excitation, as well as the presence of harmonics. One of the main original contributions is to share the data sets to give the opportunity to researchers for testing and validating analytical or numerical models of a nonlinear beam with non-ideal boundary conditions and subjected to one stochastic broadband excitation. This Data in Brief article is an additional item directly alongside the following paper published in the Communications in Nonlinear Science and Numerical Simulation (CNSNS) journal: T. Roncen, J-P. Lambelin and J-J. Sinou, *Nonlinear vibrations of a beam with non-ideal boundary conditions and stochastic excitations - experiments, modeling and simulations,* Communications in Nonlinear Science and Numerical Simulation,74 (2019) 14-29. doi.org/10.1016/j.cnsns.2019.03.006

## Specifications Table

**Table:** 

Subject	Mechanical engineering
Specific subject area	Structural vibration, stochastic broadband excitation, nonlinear vibration, CEA-beam benchmark structure
Type of data	Tables, figures, *.mat files and *.txt ASCII files
How data were acquired	All the dynamic signal generation and acquisition are performed via the data acquisition platform Simcenter Testlab software from Siemens, that controls the timing, synchronization, and data transfer between the Sensor-Based Input/Output modules and the external host.
Data format	Raw and analyzed data
Parameters for data collection	Investigation of nonlinear vibrations for the CEA-beam benchmark structure to one stochastic broadband excitation around [115;135] Hz for four RMS levels of excitation: 0.4 m.s^-^^2^, 2 m.s^-^^2^, 4 m.s^-^^2^ and 8 m.s^-^^2^.
Description of data collection	The data give input and output Power Spectral Density (PSD) for the accelerometer at the center of the beam for four different RMS level of excitation.
Data source location	Data obtained from the CEA/CESTA laboratory, CS60001, 15 avenue des Salinières, 33116 Le Barp, France
Data accessibility	The data are available in this article as a supplementary files
Related research article	This data is supplementary to article ‘Nonlinear vibrations of a beam with non-ideal boundary conditions and stochastic excitations - experiments, modeling and simulations’, Communications in Nonlinear Science and Numerical Simulation, 74 (2019) 14-29. doi.org/10.1016/j.cnsns.2019.03.006

## Value of the Data

• The database provides the nonlinear response measurements at the center of the beam for the CEA-beam benchmark structure subjected to one stochastic excitation for four levels of excitation: 0.4 m.s^−2^, 2 m.s^−2^, 4 m.s^−2^ and 8 m.s^−2^.
• The data could be useful for researchers and industrial in understanding of the nonlinear bending behavior of a beam subjected to one stochastic excitation: the enlargement of the response peak in the vicinity of the primary resonance, as well as the appearance of secondary peaks resulting from the harmonics generated by the primary resonance.
• The database gives the opportunity to researchers for comparing and validating analytical and numerical models for predicting the nonlinear dynamic behavior (within a frequency range of interest [50;1000] Hz) of a clamped–clamped steel beam with non-ideal boundary conditions and subjected to one stochastic excitation.
• This database enriches the previous open database proposed as part of the work on the nonlinear behavior of the experimental CEA-beam benchmark structure subjected to low and high levels of sinus excitation signal [1], [2].

## 1 Data Description

This dataset is provided as supplementary data in a Matlab format *.mat and ASCII format *.txt.

The data are provided as follows: “data_04RMS.mat”, “data_2RMS.mat”, “data_4RMS.mat” and “data_8RMS.mat” (“data_04RMS.txt”, “data_2RMS.txt”, “data_4RMS.txt” and “data_8RMS.txt” respectively) give the input and output PSD for the four RMS levels of excitation: 0.4 m.s^−2^, 2 m.s^−2^, 4 m.s^−2^ and 8 m.s^−2^ in the Matlab format (ASCII format, respectively). Description of the columns headings is provided in Table 1.

**Table 1:** Description of the dataset for *.txt and *.mat files

Column	Signal	Quantity	Unit
1	Input	Frequency	Hz
2	Input	Input PSD	g^2^/Hz
3	Output	Output PSD in A1	g^2^/Hz

**Fig. 1:**
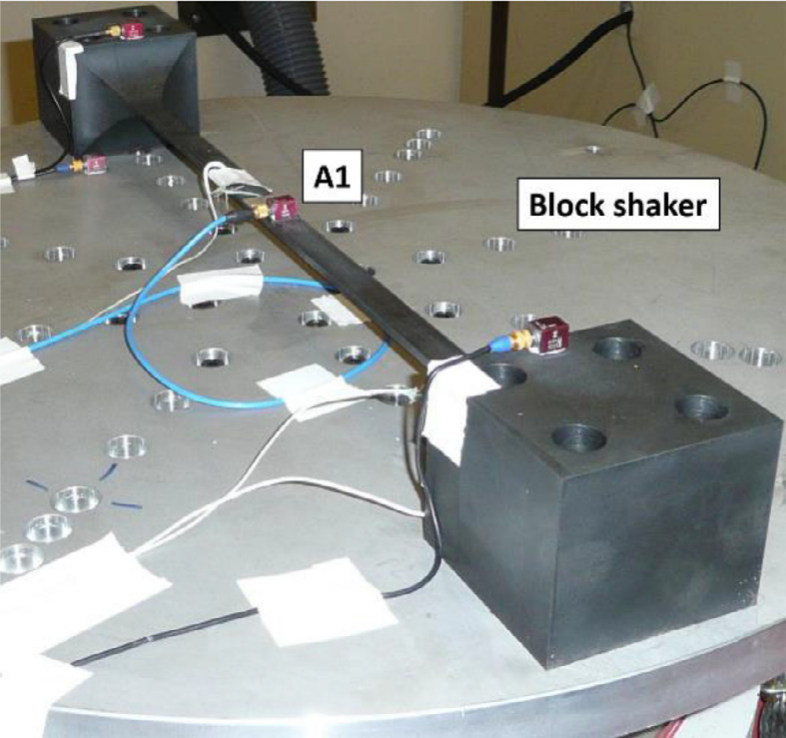
Experimental setup of the CEA-beam benchmark structure [1], [2], [3]

Each provided data corresponds to the vertical displacement at the center of the beam (see the position A1 in Fig. 1). Acquisition of the experimental outputs are performed by using a miniature triaxial piezoelectric accelerometer with integral hybrid electronics (Ref. Endevco model 66M5).

## 2 Experimental Design, Materials and Methods

The CEA-beam benchmark structure is a clamped–clamped steel beam with non-ideal boundary conditions. It consists of a beam and two blocks made from a single piece of steel as shown in Fig. 1. The two blocks are bolted onto a large circular aluminum plate, itself bolted onto the vibrating pot. The technical drawing of the CEA-beam benchmark structure is given in [2]. All the physical parameters are also given in [3].

The following paragraph briefly describes the experimental protocol. As described in [3], the shaker is piloted with a Power Spectral Density (PSD) of various levels of excitation (i.e. four RMS levels of 0.4 m.s^−2^, 2 m.s^−2^, 4 m.s^−2^ or 8 m.s^−2^). The sampling frequency and the resolution frequency are 12800 Hz and 0.39 Hz, respectively. The output signal is decomposed into 100 temporal blocks of 2.62 s each. A periodogram estimate computes an approximation of the PSD for each of the 100 blocks. Then, the PSD of the output signal is calculated by averaging all the estimates. The input PSD is constant over time to ensure that the response is stationary. The input PSD is centered around the frequency of the primary resonance (i.e [115;135] Hz as seen in [3]). It is constant over time to ensure that the output response is stationary. It can be noted that the dynamic behavior of the reference experimental structure of the CEA beam subjected to harmonic excitations has been previously performed in [1], [2] which allows us to identify the value of the first resonance frequency.

**Fig. 2:**
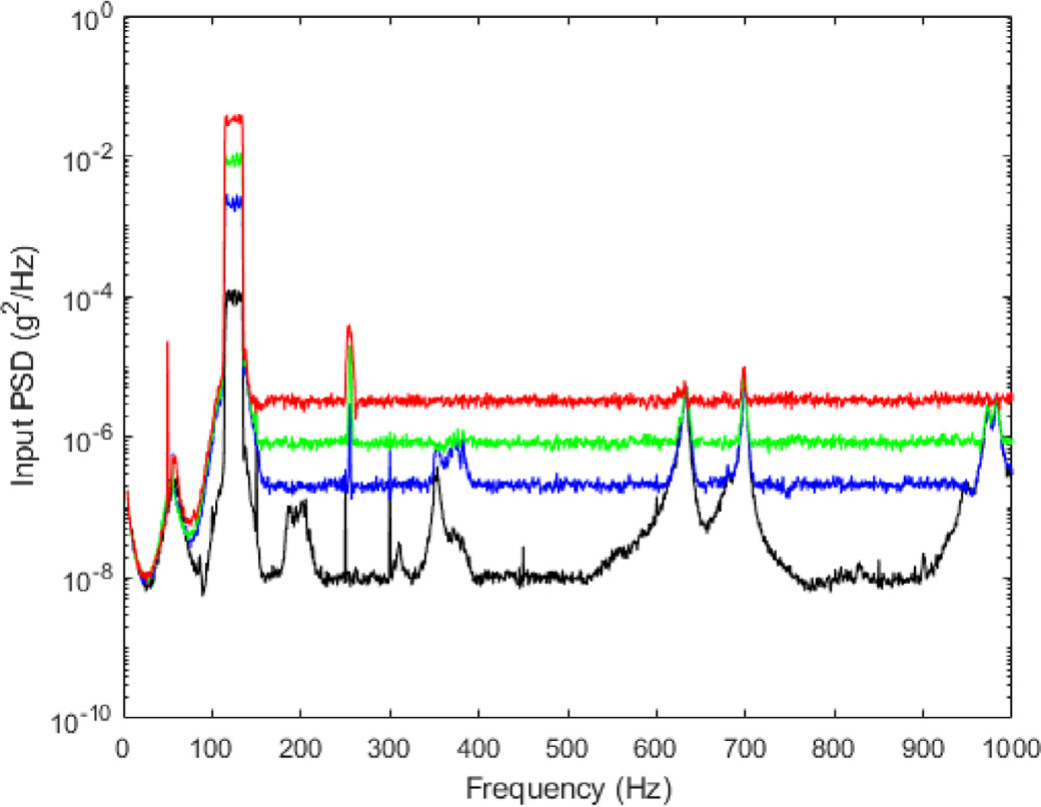
Input experimental PSD for the experiment with a constant RMS level of 0.4 m.s^−2^ (black), 2 m.s^−2^ (blue), 4 m.s^−2^ (green) and 8 m.s^−2^ (red)

**Fig. 3:**
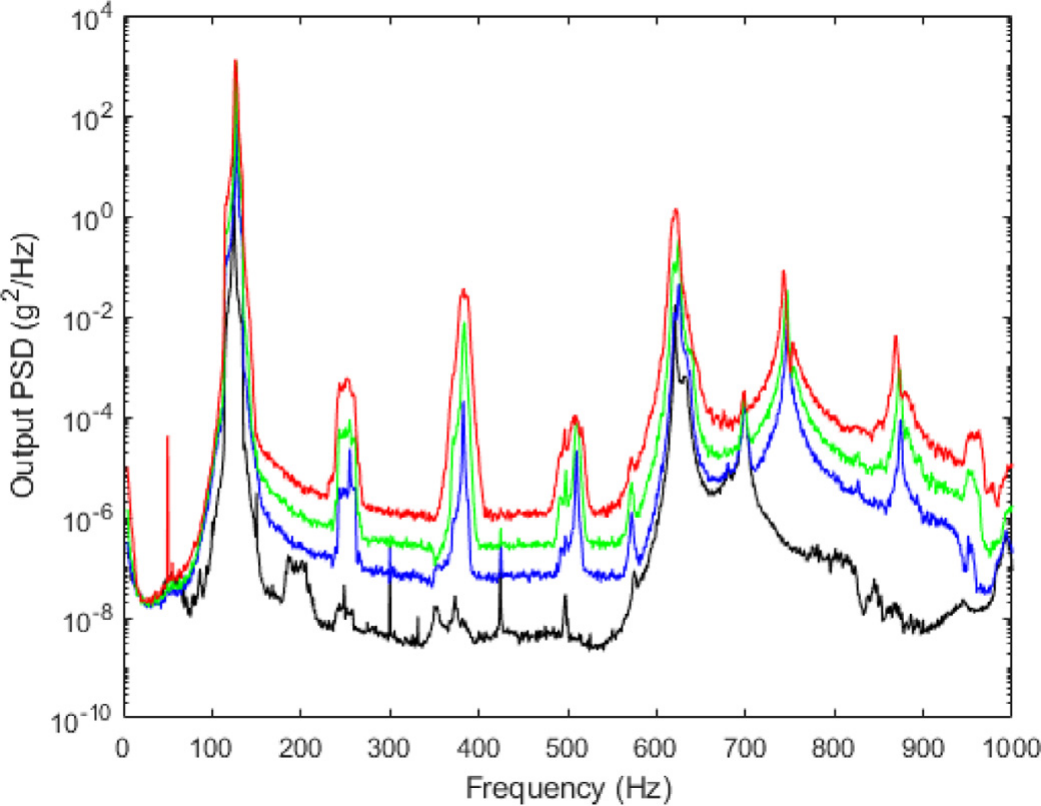
Output experimental PSD of accelerometer A1 for the experiment with a constant RMS level of 0.4 m.s^−2^ (black), 2 m.s^−2^ (blue), 4 m.s^−2^ (green) and 8 m.s^−2^ (red)

Despite on the fact that the previous study scientific analysis [3] was carried out only for the frequency range [50;500] Hz, the frequency range of interest has been extended to [50;1000] Hz for the open data provided. This gives the opportunity to researchers for conducting additional analysis compared to [3] and the development of extended analytical and numerical models for predicting the nonlinear dynamic behavior within a frequency range of interest [50;1000] Hz.

Input and output experimental results for the four levels of excitation are plotted in Fig. 2 and Fig. 3, respectively. All results on the frequency range [50;500] Hz have been previously analyzed and discussed in [3] for characterization of the nonlinear behavior of the CEA-beam structure beam subjected to a broadband random excitation. As previously explained in [3], the presence of noise in the bandwidth at low and medium excitation levels (around [185;205] Hz, [350;390] Hz, [600;720] Hz and [940;1000] Hz) as well as the appearance of the undesirable resonance peak in the input around 250 Hz illustrates the limitation of the control of the input PSD.

## CRediT Author Statement

**T. Roncen:** Conceptualization, Methodology, Validation, Investigation, Resources, Writing Review & Editing; **J.-P. Lambelin:** Conceptualization, Methodology, Validation, Writing Review & Editing, Supervision, Project administration, Funding acquisition; **Y. Chantereau:** Validation, Investigation, Resources; **J-J. Sinou:** Conceptualization, Methodology, Validation, Investigation, Writing Original Draft, Writing Review & Editing, Visualization, Supervision, Project administration.

## Declaration of Competing Interest

The authors declare that they have no known competing financial interests or personal relationships which have or could be perceived to have influenced the work reported in this article.

## Appendix Supplementary materials

Image, application 1
